# Hemoptysis With Expulsion of Coil Fragments in a Patient With Aorto‐Bronchial Fistula of the Thoracic Descending Aorta

**DOI:** 10.1002/ccr3.71276

**Published:** 2025-12-04

**Authors:** Lorenzo Giovannico, Domenico Parigino, Giuseppe Fischetti, Federica Mazzone, Luca Savino, Claudia Leo, Giuseppe Cristiano, Aldo Domenico Milano, Tomaso Bottio

**Affiliations:** ^1^ Cardiac Surgery Unit, Department of Precision and Regenerative Medicine and Ionian Area (DiMePRe‐J) University of Bari Medical School Bari Italy

**Keywords:** aortic surgery, Aorto‐bronchial fistula, coil migration, hemoptysis, TEVAR, thoracic aorta

## Abstract

Massive hemoptysis following TEVAR, especially in patients with prior coil embolization, should raise immediate suspicion for aortobronchial fistula. This rare but life‐threatening condition demands prompt imaging, bronchoscopy, and often urgent surgical repair. Early multidisciplinary collaboration significantly improves prognosis.

## 1 Introduction

Aortobronchial fistula (ABF) is a rare but potentially fatal condition characterized by an abnormal communication between the thoracic aorta and the bronchial tree. While uncommon, ABF is a recognized complication following thoracic endovascular aortic repair (TEVAR), with an incidence ranging from 1.5% to 1.9% [1]. Hemoptysis, varying from intermittent to massive, is the most frequent presenting symptom and may be the sole clinical manifestation prior to catastrophic hemorrhage [2].

The pathogenesis of ABF post‐TEVAR involves several mechanisms, including erosion of the aortic wall due to persistent endoleaks, infection, or mechanical injury from stent grafts or embolization coils [3]. Coil migration into the bronchial system, though exceedingly rare, has been documented and can directly contribute to fistula formation [4]. Early diagnosis is paramount, as ABF carries a high mortality rate if left untreated. Computed tomography angiography (CTA) serves as the initial imaging modality of choice, offering valuable information on aortic pathology and potential fistulous connections. However, bronchoscopy remains the most sensitive and specific method to identify the bleeding site, allowing direct visualization of the fistulous tract or intrabronchial foreign bodies such as coils [5, 6, 7, 8]. Management strategies for ABF depend on the patient's hemodynamic stability and the extent of the fistula. TEVAR offers a less invasive option and can be effective in selected cases, particularly in high‐risk surgical candidates. Nevertheless, open surgical repair remains the definitive treatment, especially in cases complicated by infection, graft failure, or recurrent fistula formation [9].

In this report, we present the case of a 67‐year‐old woman who developed an ABF with intrabronchial coil migration following TEVAR and coil embolization for a type II endoleak. The patient was successfully treated with emergency open surgical repair, highlighting the importance of prompt recognition and a multidisciplinary approach in managing this life‐threatening complication.

## 2 Case History/Examination

A 67‐year‐old woman with a history of thoracic endovascular aortic repair (TEVAR) performed 3 years prior for a descending thoracic aortic aneurysm presented to the emergency department with sudden onset of massive hemoptysis. On arrival, she was hypotensive (blood pressure: 85/50 mmHg), tachycardic (heart rate: 110 bpm), and hypoxic (SpO_2_: 88% on room air). Physical examination revealed decreased breath sounds over the left lung field. Laboratory tests showed a hemoglobin level of 8.2 g/dL, indicating significant blood loss.

## 3 Differential Diagnosis, Investigations, and Treatment

**FIGURE 1:**
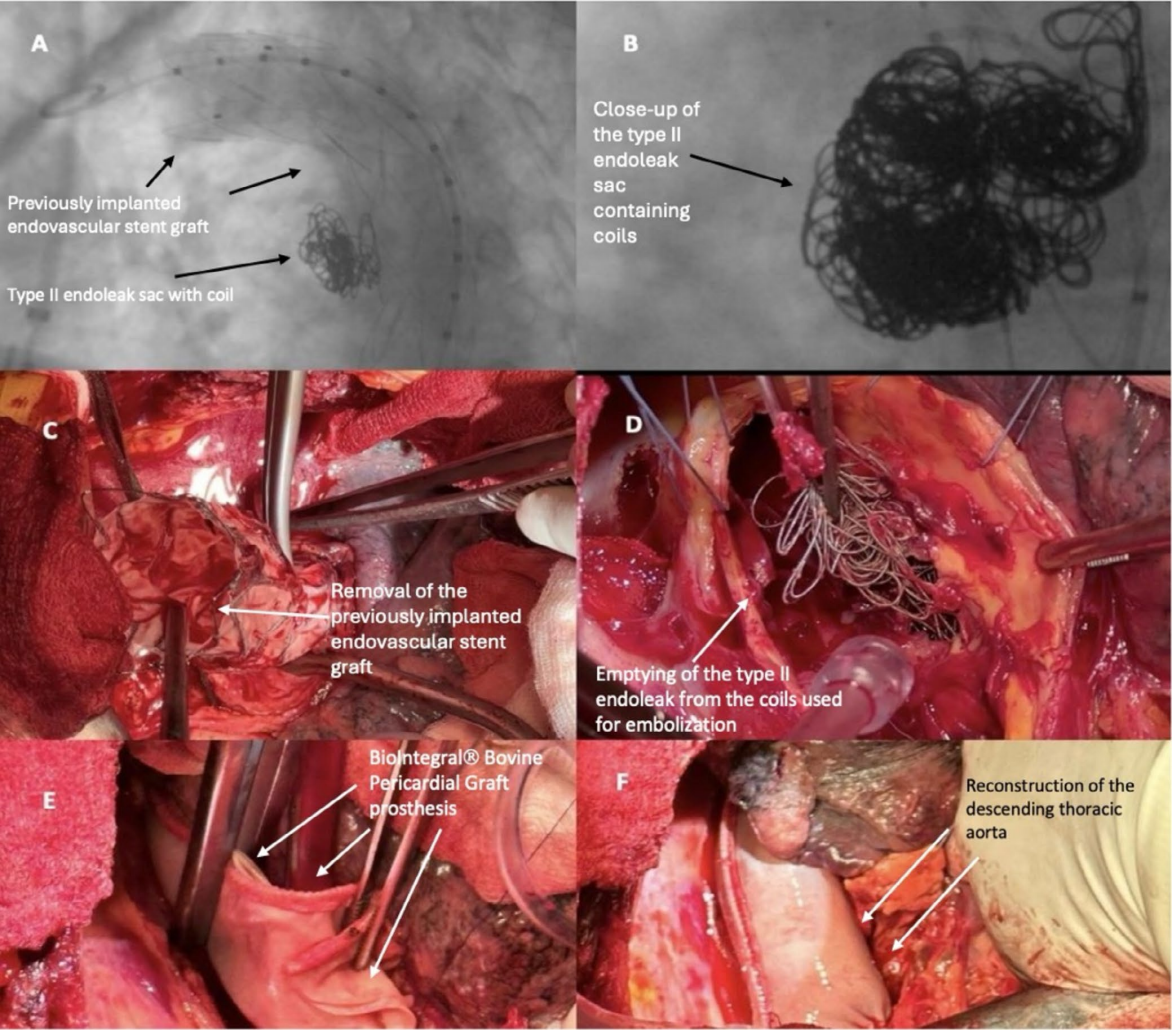
(A) Aortography showing the type II endoleak sac (arrow 1), the previously implanted endovascular stent graft (arrow 2), and the embolization coils (arrow 3). (B) Close‐up view of the type II endoleak sac with embolization coils (arrow). (C) Removal of the previously implanted endovascular stent graft (arrow). (D) Extraction of coils from the type II endoleak sac (arrow). (E) Reconstruction of the descending thoracic aorta with a BioIntegral bovine pericardial graft (arrow). (F) Final result showing reconstruction of the descending thoracic aorta and closure of the aortobronchial fistula (arrow).

Given the patient's presentation, differential diagnoses included pulmonary embolism, bronchiectasis with bleeding, lung malignancy, and aortobronchial fistula. A contrast‐enhanced computed tomography (CT) scan of the chest demonstrated a type II endoleak from the left subclavian artery with evidence of coil migration (AZUR CX Coil (Terumo)) into the bronchial tree and a suspected aortobronchial fistula (ABF) (Figure 1A,B). Bronchoscopy confirmed the presence of metallic coils protruding into the left main bronchus, consistent with coil migration and fistula formation. Given the patient's hemodynamic instability and the risk of ongoing hemorrhage, emergent surgical intervention was undertaken. Intraoperatively, the previously implanted endoprosthesis (Figure 1A,C) and the migrated coils were removed (Figure 1A,D). The aorta was subsequently reconstructed with a BioIntegral Bovine Pericardial Graft prosthesis (BioIntegral Surgical Inc.) (Figure 1A,E,F) and the integrity of the bronchus was re‐established with a PTFE patch. The bronchial injury was addressed with primary repair reinforced with a pedicled intercostal muscle flap to prevent recurrence. Postoperatively, the patient was managed in the intensive care unit with mechanical ventilation and broad‐spectrum antibiotics.

## 4 Conclusion and Results (Outcome and Follow‐Up)

She was weaned off ventilatory support on postoperative Day 5 and discharged home in stable condition on postoperative Day 14. At the 6‐month follow‐up, she remained asymptomatic with no evidence of recurrent fistula or endoleak on imaging studies.

## 5 Discussion

Aortobronchial fistula (ABF) is a rare but potentially fatal complication that can occur after thoracic endovascular aortic repair (TEVAR), with an estimated incidence of 1.5%–1.9% [1]. It is most commonly caused by erosion of the aortic wall due to persistent endoleaks, mechanical trauma from oversized stent grafts, infection, or—in rare cases—coil migration following embolization procedures [1, 2, 3]. Hemoptysis, ranging from mild to massive, is the hallmark symptom and often represents the only warning sign before catastrophic hemorrhage [1, 4].

Diagnosis can be challenging, and imaging plays a central role. Contrast‐enhanced CT and CT angiography are commonly used for initial evaluation, revealing suggestive signs such as periprosthetic gas, hematoma, or coil displacement [1]. However, bronchoscopy remains the most sensitive method to identify the bleeding site and may directly visualize the fistulous tract or foreign material protruding into the bronchial lumen [1, 5].

Management strategies depend on the clinical status of the patient. In hemodynamically stable patients with favorable anatomy, TEVAR may be used to exclude the fistula. However, in cases with infection, graft failure, or persistent bleeding—as in our patient—open surgical repair remains the treatment of choice [2, 9]. Surgical approaches typically involve resection of the diseased aortic segment and airway reconstruction using vascular grafts and tissue patches. In our case, removal of the migrated coils, aortic replacement with a bovine pericardial graft, and bronchial repair using a PTFE patch resulted in full recovery.

The literature emphasizes that ABFs tend to occur earlier after TEVAR (mean 1.8 years) than after open aortic surgery or in untreated aneurysms [1]. Coil‐related fistulae, although rare, have been reported and are likely under‐recognized [3, 5]. Infections and repeated interventions further increase the risk of fistula formation and are associated with worse outcomes [1, 4, 9].

This case highlights the importance of early recognition of ABF symptoms, particularly in patients with a history of TEVAR and coil embolization. A multidisciplinary approach involving cardiovascular surgeons, pulmonologists, and interventional radiologists is essential to optimize diagnosis and improve outcomes in ABF cases. Recent reports emphasize that coordinated care reduces perioperative mortality and recurrence [8, 9].

Similar cases of aortobronchial fistula following TEVAR and coil embolization have been reported in the literature, albeit rarely. For instance, Fukuhara et al. described a case of ABF with coil migration in a patient with previous embolization for endoleak, underlining the role of persistent foreign bodies as a source of erosion and fistulization [6]. Likewise, Marone et al. emphasized the importance of early suspicion when hemoptysis occurs in patients with prior endovascular thoracic procedures [7].

More recent reports have also expanded our understanding of this rare complication. Tinica et al. described a hybrid surgical and endovascular approach for an ABF after patch aortoplasty in a patient with SARS‐CoV‐2 pneumonia, highlighting the feasibility of combined strategies [10]. Greene et al. reported massive hemoptysis from ABF occurring decades after childhood aortic coarctation repair, underlining the potential for very late presentations [11]. Similarly, Morimura et al. described transarterial embolization of the pulmonary ligament artery as a successful alternative in an elderly patient with post‐aortic repair hemoptysis [12]. These cases underscore the diverse clinical scenarios, therapeutic options, and long‐term vigilance required in patients with prior thoracic aortic interventions.

In comparison to these reports, our patient presented with a more acute onset and massive bleeding, necessitating immediate surgical intervention. These similarities reinforce the need for heightened clinical vigilance, particularly when coil migration is identified on imaging. Furthermore, the literature supports the view that open surgical repair is preferable when there is active infection, foreign body erosion, or hemodynamic instability [8, 9].

While TEVAR can be a valuable tool in selected patients, its role remains limited in complex ABFs, especially those involving airway compromise. The long‐term success of open surgery in such cases suggests that early referral to specialized centers is warranted when ABF is suspected.

Aortobronchial fistula (ABF) is a rare but life‐threatening complication following thoracic endovascular aortic repair (TEVAR), often presenting with hemoptysis as the sole warning sign. Early recognition and prompt intervention are crucial to prevent catastrophic outcomes. While TEVAR offers a less invasive treatment option, it may not address all underlying issues, and open surgical repair remains the definitive treatment in many cases [3, 8].

This case underscores the importance of a multidisciplinary approach and vigilant long‐term follow‐up in patients undergoing TEVAR, especially when adjunctive procedures like coil embolization are involved. Clinicians should maintain a high index of suspicion for ABF in patients presenting with hemoptysis post‐TEVAR and consider comprehensive diagnostic evaluations to facilitate timely and appropriate management [1, 5].

## Author Contributions

**Lorenzo Giovannico:** conceptualization, writing – original draft, writing – review and editing. **Domenico Parigino:** methodology, writing – original draft. **Giuseppe Fischetti:** conceptualization, validation, visualization. **Federica Mazzone:** supervision. **Luca Savino:** data curation, validation, visualization. **Claudia Leo:** data curation, visualization. **Giuseppe Cristiano:** formal analysis, visualization. **Aldo Domenico Milano:** conceptualization, supervision, writing – review and editing. **Tomaso Bottio:** data curation, formal analysis, supervision, writing – review and editing.

## Consent

Written informed consent was obtained from the patient for publication of this case report and accompanying images.

## Conflicts of Interest

The authors declare no conflicts of interest.

## Data Availability

The data that support the findings of this study are available from the corresponding author upon reasonable request.
